# The influence of Tai Chi exercise on the subjective well-being in the aged: the mediating role of physical fitness and cognitive function

**DOI:** 10.1186/s12877-023-04366-3

**Published:** 2023-10-09

**Authors:** Heng Wang, Yangyang Liu, Zhengguo Pei, Jiafeng Liang, Xiaosheng Ding

**Affiliations:** 1 College of Physical Education, Henan Normal University, Xinxiang, 453007 China; 2 Faculty of Education, Henan Normal University, Xinxiang, 453007 China

**Keywords:** Aged, Tai Chi exercise, Subjective well-being, Physical fitness, Self-control, Executive function

## Abstract

This study investigated the effects of Tai Chi exercise on subjective well-being in the aged. The participants were randomly assigned to an experimental group or a control group. The experimental group received 12-week Tai Chi exercise while the control group maintain their original living habits. The participants’ subjective well-being, physical fitness, self-control, and executive function were measured at baseline and after 12 weeks of Tai Chi exercise. Results: (1) Tai Chi exercise can positively affect the subjective well-being of the aged (F_(1,78)_ = 37.699, p < 0.001); (2) Tai Chi exercise could affect the subjective well-being in the aged through the independent intermediary of physical fitness (95% CI=[0.115, 0.485]) and self-control (95% CI=[0.109, 0.433]); (3) Tai Chi exercise could indirectly affect the subjective well-being in the aged through the chain mediation of executive function and self-control (95% CI=[0.009, 0.104]). This study provides valuable insights into the potential benefits of Tai Chi exercise for subjective well-being in the aged.

## Introduction

Subjective Well-being (SWB) is the overall evaluation and emotional experience of life from an individual’s perspective, including life satisfaction, depressive symptoms, psychological well-being, and affective well-being. It is an essential indicator of measuring the quality of life [1] and is considered a key priority in the aged population for promoting healthy aging [2]. A number of studies have shown that SWB reflects an individual’s mental health, that it is a holistic assessment of the evaluator’s own quality of life based on certain self-defined criteria, that it is a subjective reflection of objective reality, and that it is a key factor affecting the quality of life of the aged [3–6], because it can prevent the development of certain psychopathological and physical health problems [7]. Some scholars [8, 9] considered SWB as a standard for health evaluation. Thus, in the context of global aging, investigating ways to improve the subjective wellbeing of the aged holds significant theoretical and practical significance for promoting healthy aging.

Physical activity or exercise has always been regarded as one of the important lifesty le factors to maintain the physical and mental health of the aged [10]. It is also a low-cost and effective intervention method for improving physical fitness and cognitive function, which can effectively enhance the quality of life of the aged and alleviate health inequalities caused by economic conditions [11]. According to Akbari [12], moderate supplementation with micronutrients such as vitamin B12 and folic acid can help improve cognitive function and increase physical activity in older adults.Vancini [13] found that, in the long run, ultra-endurance exercise, high-volume physical activity with moments of peak intensity (six hours or more), was associated with the risk of heart disease, sudden death, telomere shortening, accelerated cellular senescence, and impairment of the risk of healthy aging processes and longevity. And some studies have argued that cognitive and physical training have positive psychophysiological effects on older adults [14–16]. Although previous studies have explored the relationship between physical exercise and SWB, the mechanism by which physical exercise affects SWB is still lacking and inadequate due to different intervention methods and research perspectives [17, 18]. This study is mainly based on the efficacy of physical and mental exercise, which improves the physical health and cognitive function of the body by focusing attention and controlling breathing while engaging in physical activities, such as Tai Chi, yoga, Qigong, etc. [19, 20]. Zou L et al. [21] and Zhou S et al. [22] analyzed the effects of different styles of Tai Chi exercises, with a particular emphasis on the impact of Chen style Tai Chi on the aged. Therefore, this study chose Chen style Tai Chi as an intervention method to explore how Tai Chi exercise can improve the SWB of the aged from the perspective of their physical and cognitive health.

## Literature review and research hypothesis

### The relationship between physical exercise and SWB

Previous studies have found a positive dose-response relationship between physical exercise and SWB. For instance, Richards et al. [23] discovered that, compared to inactive individuals, those who were insufficiently active, sufficiently active, and very active had a 20%, 29%, and 52% higher likelihood of being happy, respectively, based on a survey of citizens in 15 European countries. Recently, a meta-analysis conducted by Buecker et al. [24] demonstrated that 82.24% of physical exercise interventions in 157 studies had a positive impact on SWB, and individuals who engaged in physical exercise reported significantly higher scores of SWB than those who did not. Moreover, from a neurobiological perspective, this causal effect can be indirectly inferred. For example, the hypothalamic-pituitary-adrenal axis, the response of brain-derived neurotrophic factor, and the “endorphin hypothesis” all indicate that physical activity can reduce depression and anxiety [25–27]. These findings suggest that physical exercise can indeed improve an individual’s SWB. Therefore, we propose hypothesis 1 of this study that Tai Chi exercise has a positive effect on SWB in the aged.

### The role of physical fitness in the relationship between physical exercise and SWB

Many studies have found that physical fitness has a significant impact on an individual’s SWB [28, 29]. For example, Huang et al. [24] found that good physical health, that is, good physical fitness, can effectively enhance people’s subjective well-being. Moreover, study [17] from a biological perspective has found that physical exercise can stimulate the exercise organs to improve physical fitness, thereby promoting individual happiness experiences.

For the aged, with aging and prolonged sedentary behavior, their physical health gradually declines, manifested in weakened muscle strength, endurance, flexibility, and balance abilities [30, 31]. These declines in physical function significantly impact their daily living activities, increasing their risk of falls and injuries, which are the main cause of their morbidity and mortality, ultimately leading to a decrease in their SWB. Some studies [32–34] have found that through appropriate physical exercise, aged people can increase their muscle strength, aerobic capacity, and bone density, thereby restoring their defects in strength and muscle quality to improve their physical fitness, and ultimately improving their quality of life [35, 36].

To summarize, physical exercise first improves the physical fitness of the aged, and then further enhances their SWB. Thus, we propose hypothesis 2: physical fitness mediates the relationship between Tai Chi exercise and SWB in the aged.

### The role of cognitive function in the relationship between physical exercise and SWB

The decline in cognitive function caused by aging is mainly manifested in a decline in self-control and executive function. Self-control refers to the process or behavior of resisting temptation or prioritizing (simultaneous or long-term) goal attainment [37]. Recently, a team of 24 Chinese experts from different fields reviewed researches about the relationship between physical exercise and cognitive decline in the aged, and reached a consensus [38] that physical exercise has a positive impact on the cognitive function of the aged, particularly in the areas of overall cognitive function, memory, self-control, and executive function.

Furthermore, psychological research has shown that increasing self-control level can promote individual’s SWB. For example, individuals with high self-control have lower depression, while individuals with low self-control experience higher depression [39]. Individuals with higher self-control levels tend to adopt more effective strategies to achieve goal, thus experience longer-term happiness [40]. Higher self-control can bring multiple positive life outcomes for individuals, such as physical health, interpersonal relationships, and work/study achievements, all of which have an impact on SWB. Therefore, we propose hypothesis 3 of this study: Self-control plays a mediating role in the process of Tai Chi exercise affecting SWB in the aged.

Executive function refers to the ability to plan, initiate, sequence, and monitor complex goal-oriented behaviors, as well as to control complex activities [41]. It is crucial for an individual’s independent daily life and behavioral adaptation. The decline in executive function can lead to amnesia, depression, and low self-esteem [42, 43], and increase the risk of mild cognitive impairment and Alzheimer’s disease in older adults [44, 45], which impose a serious burden on personal life, family, and society, thereby reducing the quality of life and SWB of older adults. Additionally, researches [46, 47] have found that individuals’ executive function affects their self-control ability. Neuroscience research [48] also provided evidence for this, suggesting that low self-control is the result of executive dysfunction. Therefore, individuals’ executive function affects the development of their self-control.

**Fig. 1:**
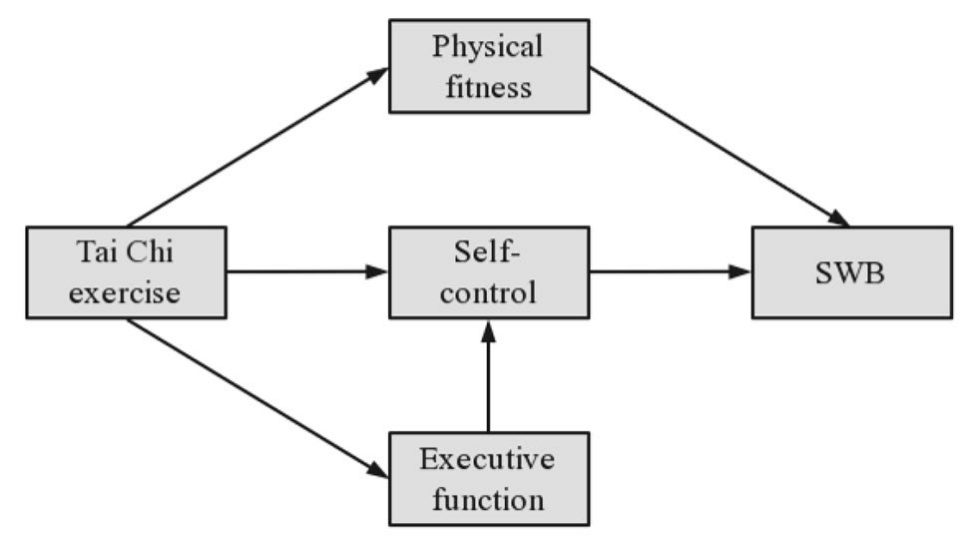
Hypothesis model for this study

Based on the above analysis, physical exercise can improve the cognitive function, especially self-control and executive function of the aged. And the enhancement of these cognitive components further improves SWB of the aged. Additionally, the development of executive function can promote self-control. Therefore, hypothesis 4 is proposed: Executive function and self-control play a chain mediating role between physical exercise and SWB. And based on this, a hypothesis model of this study is constructed (see Fig. 1).

## Methods

### Participants

The G * power 3.1.9 software was used to estimate the sample size. Effect size = 0.25, α error = 0.05. It was calculated that the statistical power of 0.95 can be reached with 54 subjects.

Aged 60–69 years old (n = 80) who had no Tai Chi practice experience in Zhoukou City, Henan Province were selected, and randomly divided into experimental group (n = 40) and control group (n = 40) using SPSS 27.0 “Visual Binning”. Inclusion criteria: ①The aged’s family and themselves all agreed to participate; ②The aged is able to complete normal physical exercise; ③No psychotropic drugs or major surgery.

The aged in experimental group underwent Chen’s 24-style Tai Chi exercise, 4 times a week (60 min for each time) for a total of 12 weeks. The aged in control group maintained their original living habits.

### Designs and measurements

The experimental design of 2 (group: experimental and control group) × 2 (time: before and after the experiment) was adopted, and the following indexes were measured.

(1) Subjective Well-being: We used General Well-Being Schedule [49] (Chinese version) which was originally developed by the National Center for Health Statistics of the United States as a type of measurement tool, and was localized modified by Chinese scholar Duan Jianhua in 1996. There are 18 items in this scale. Cronbach’s α coefficient of the scale is 0.91. In this study, SWB of the aged were evaluated by the General Well-Being Scale because Buecke et al. [24] found that the association between physical activity and SWB did not depend on the specific domain of SWB, and physical activity was equally correlated with health-specific well-being and general well-being or other domains of well-being.

(2) Self-control: The “Aged people’s Sense of Self-control Questionnaire” [50] was used to test the self-control of participants. The questionnaire consisted of 15 items and was scored with 5 points. Cronbach’s α coefficient was 0.88.

(3) Executive function: The tests of the three sub-functions of executive function were programmed by E-Prime 2.0 software. All participants completed the 3 tests in turn, and each test had its corresponding practice-trials. The formal test was entered when practice accuracy was ≥ 80% (the average accuracy of the 3 tests was 82.6%, 85.3% and 81.8%, respectively).

①Inhibitory function: Stroop task [51] was used for testing inhibitory function. The experiment consisted of a color judgment task and a word judgment task. In the formal experiment, the subjects were asked to do the task of word meaning judgment or color judgment. The subjects pressed any key to formally start the experiment. In the test task, “+” appeared in the center of the screen for 500ms, and then appeared the stimulus of “red” or “green” for 1500ms, which was divided into two forms: consistent condition and inconsistent condition. Consistency condition, the color and meaning of two Chinese characters are the same (for example, the “green” character shows green, and the “red” character shows red); Inconsistent conditions, Chinese characters with different colors and meanings (e.g., “green” characters in red, “red” characters in green). When Chinese characters appear, two forms of judgment are performed. The “green” character is shown in green and the “red” character is shown in red. Press F key; “Green” is displayed in red and “red” is displayed in green. Press J. The sequence of consistent and inconsistent stimuli appeared randomly, with 60 trials each. The interval of stimulation was 1000ms. The test score is the mean response time of the inconsistent condition minus the mean response time of the consistent condition. The smaller the difference, the better the inhibitory ability. Require accuracy and speed.

②Updating function: N-back task [52] is adopted. Perform 2 tasks, 1-back: A number from 1 to 9 will appear on the computer screen, and each number will be presented separately in the center of the computer screen. The presentation time of the stimulus number will be 800ms, and the stimulation interval will be 3000ms. The participants are required to look at these numbers carefully. 2-back: A number from 1 to 9 will appear on the screen, and each number will be presented separately in the center of the computer screen. The presentation time of the stimulus number is 1000ms, and the stimulation interval is 3000ms. The subjects are required to look at these numbers carefully. There were 60 trials. The test score is the average reaction time. The smaller the reaction time (ms), the better the updating ability. Require accuracy and speed.

③Shifting function: More Odd Shifting task [52, 53] was used to test. A series of numbers were presented one by one in the center of the computer screen, with a time of 3000ms and a stimulation interval of 3000ms. The subjects judged the numbers (1–9 excluding 5) according to the requirements. The computer performed three tasks: Task A, large/small judgment: red numbers were presented, and if they were less than 5, they would press the “F” key to respond; If more than 5, press the “L” key reaction. B task: odd/even judgment, show black numbers, if odd, press “F” key reaction; If it is even, press the “L” key to react. C Task: Big/small - odd/even judgment, present the number if it is red, make “big/small” judgment; If it is black, make an “odd/even” judgment. Task A and Task B do not need to convert 16trials each, and Task C needs to convert 32trials. The final test score is the average reaction time of the shifting task minus the average reaction time of the corresponding non-shifting task. The smaller the reaction time difference value, the better the shifting ability. Require accuracy and speed.

(4) Physical fitness test: Strength (grip strength) was measured using grip meter (GMCS-WCS3). Flexibility was tested by seated flexion (GMCS-TQQ3). Balance ability was measured by standing on one foot with eyes closed (GMCS-DJZL3). Endurance was measured by using spirometer (GMCS-FHL3) [54].

### Mathematical statistics method

SPSS 27.0 was used for statistical analysis of the collected data, and the fitting of the model was set and tested in the software spssAmos28.

## Results

### Baseline assessment

Independent sample t test was conducted for all variables of the two groups before exercise intervention to assess the baseline of the two groups. The results showed that the both groups had no significant differences in other variables except for the endurance (t=-2.30, p = 0.027) (see Table 1), which indicates that the psychological and physical indicators of the two groups at the same level before intervention.

**Table 1:** Baseline assessment results and descriptive statistics of the two groups at pre- and post-intervention (M, SD)

Variables	Dimensions	Pre-test		*t*	*P*	Post-test	
		experimental group	control group			experimental group	control group
EF	Inhibitory (ms)	888.25 (78.28)	893.95 (55.09)	0.26	0.794	809.20 (80.85)	881.35 (51.15)
	Updating (ms)	941.60 (37.11)	946.15 (40.44)	0.37	0.716	872.95 (33.55)	943.80 (45.76)
	Shifting (ms)	837.55 (88.15)	833.75 (102.66)	-0.12	0.902	824.25 (76.61)	818.35 (70.85)
PF	Endurance (ml)	2305.25 (428.49)	2591.35 (343.65)	-2.30	0.027	2363.50 (371.34)	2649.20 (370.88)
	Strength (kg)	21.175 (4.15)	21.22 (2.26)	-0.14	0.887	25.56 (4.46)	21.80 (2.07)
	Flexibility (cm)	-2.20 (2.35)	-2.60 (2.98)	0.47	0.644	-1.60 (3.37)	-2.75 (4.01)
	Balance (s)	8.25 (3.40)	9.00 (2.24)	-0.81	0.422	14.80 (3.12)	9.05 (1.71)
SC		40.45 (5.03)	43.35 (4.43)	-1.91	0.064	63.85 (5.32)	44.80 (4.34)
SWB		35.05 (8.98)	33.85 (8.14)	0.44	0.664	42.30 (5.46)	31.65 (9.52)

Note: EF = executive function; PF = physical fitness; SC = Self-control (the same below)

### Analysis of intervention effect of Tai Chi exercise

In order to test the intervention effect of Tai Chi exercise, 2 (groups: experimental and control group) × 2 (times: before and after intervention) repeated measurement analysis of variance were used. The descriptive statistical results of each variable are shown in Table 1 and the ANOVA results in Table 2.

**Table 2:** The results of repeated analysis of measurement variance for each variable

Variables	Time Main effect			Group Main effect			Interaction		
	F_(1,78)_	*P*	${{\eta }}_{\text{p}}^{2}$	F_(1,78)_	*P*	${{\eta }}_{\text{p}}^{2}$	F_(1,78)_	*P*	${{\eta }}_{\text{p}}^{2}$
SWB	3.330	< 0.001	0.130	24.649	< 0.001	0.240	11.662	< 0.001	0.130
SC	486.656	< 0.001	0.862	78.224	< 0.001	0.501	379.698	< 0.001	0.830
Inhibitory	27.331	< 0.001	0.257	9.960	< 0.005	0.113	14.367	< 0.001	0.156
Updating	29.853	< 0.001	0.277	39.821	< 0.001	0.338	26.032	< 0.001	0.250
Shifting	4.685	< 0.05	0.057	0.073	> 0.05	0.001	0.025	> 0.05	0.000
Strength	22.614	< 0.001	0.225	11.232	= 0.001	0.126	13.220	< 0.001	0.145
Balance	89.909	< 0.001	0.535	25.594	< 0.001	0.247	87.206	< 0.001	0.528
Endurance	13.175	< 0.01	0.145	11.747	= 0.01	0.131	0.000	> 0.05	0.000
Flexibility	0.199	> 0.05	0.657	2.242	> 0.05	0.028	0.553	> 0.05	0.007

#### Analysis of intervention effect of Tai Chi exercise on SWB

The results of ANOVA revealed that the interactions between time and groups are significant for SWB in the aged. Simple effect analysis was further conducted on the SWB, and it was found that after intervention, the SWB (F_(1,78)_ = 37.699, p < 0.001, ${{\eta }}_{\text{p}}^{2}$=0.326) of the experimental group were significantly higher than those of control group.

For the experimental group, SWB (F_(1,78)_ = 13.728, p < 0.001, ${{\eta }}_{\text{p}}^{2}$=0.150) before and after intervention are significant different, specifically manifested as the significantly higher scores for SWB after intervention compared to before intervention. For the control group, there are no significant differences in SWB before and after intervention (ps>0.1).

#### Analysis of intervention effect of Tai Chi exercise on cognitive function

The results of ANOVA revealed that the interactions between time and groups are significant for self-control, inhibitory and updating in the aged. Simple effect analysis was further conducted on the above variables, and it was found that after intervention, the self-control (F_(1,78)_ = 307.762, p < 0.001, ${{\eta }}_{\text{p}}^{2}$=0.798) of the experimental group were significantly higher than those of control group. Furthermore, inhibitory (F_(1,78)_=22.750, p<0.001, ${{\eta }}_{\text{p}}^{2}$=0.226) and updating (F_(1,78)_=62.359, p < 0.001, ${{\eta }}_{\text{p}}^{2}$=0.444) in the experimental group after intervention were significantly lower than those in the control group.

For the experimental group self-control (F_(1,78)_ = 863.040, p < 0.001, ${{\eta }}_{\text{p}}^{2}$=0.917), inhibitory (F_(1,78)_=40.665, p<0.001, ${{\eta }}_{\text{p}}^{2}$=0.343) and updating (F_(1,78)_=62.359, p < 0.001, ${{\eta }}_{\text{p}}^{2}$=0.444) before and after intervention are significant different, specifically manifested as the significantly higher scores for self-control after intervention compared to before intervention, aged while scores of inhibitory and updating being significantly lower than before intervention. For the control group, there are no significant differences in self-control, inhibitory and updating before and after intervention (ps>0.1). The interaction of group and time in shifting function of the aged were not significant. So no further analysis was conducted.

#### Analysis of intervention effect of Tai Chi exercise on physical fitness

The results of ANOVA revealed that the interactions between time and groups are significant for strength, and balance in the aged. Simple effect analysis was further conducted on the above variables, and it was found that after intervention, the strength (F_(1,78)_ = 23.395, p < 0.001, ${{\eta }}_{\text{p}}^{2}$=0.231) and balance (F_(1,78)_=177.105, p<0.001, ${{\eta }}_{\text{p}}^{2}$=0.694) of the experimental group were significantly higher than those of control group.

For the experimental group, strength (F_(1,78)_ = 35.208, p < 0.001, ${{\eta }}_{\text{p}}^{2}$=0.311) and balance (F_(1,78)_=177.105, p<0.001, ${{\eta }}_{\text{p}}^{2}$=0.694) before and after intervention are significant different, specifically manifested as the significantly higher scores for strength and balance after intervention compared to before intervention. For the control group, there are no significant differences in strength and balance before and after intervention (ps>0.1). The interaction of group and time in endurance and flexibility of the aged were not significant. So no further analysis was conducted.

The above results indicate that Tai Chi exercise can improve SWB, self-control, executive function (inhibitory and updating) and physical fitness (strength and balance) of the aged in the experimental group.

### Correlation test of variables

In order to verify the relationship between variables, Person correlation analysis was conducted for the data after intervention. The results showed that grip strength and standing on one foot with eyes closed respectively were significantly positively correlated with SWB, indicating that the better the physical fitness, the higher the SWB. There was a significant positive correlation between the sense of self-control and SWB. And inhibitory and updating function respectively were positively correlated with self-control (see in Table 3).

**Table 3:** Correlation matrix for variables

	Inhibitory	Updating	Shifting	Endurance	Strength	Flexibility	Balance	SC	SWB
Inhibitory									
Updating	0.129								
Shifting	-0.152	-0.157							
Endurance	-0.464**	-0.163	0.010						
Strength	0.330*	0.328*	-0.261	-0.146					
Flexibility	0.080	0.134	-0.068	-0.074	0.152				
Balance	0.401*	0.447**	0.073	-0.198	0.423**	0.252			
SC	0.532**	0.631**	0.000	-0.382*	0.386*	0.095	0.674**		
SWB	0.246	0.180	-0.046	-0.150	0.293*	0.093	0.407**	0.538**	

Note: * p < 0.05, ** p < 0.01, *** p < 0.001 (the same below)

### Hypothesis model test

A structural equation model was constructed to examine the mediating effects of physical fitness, self-control and executive functions on the relationship between Tai Chi exercise and SWB, with Tai Chi exercise as the independent variable and SWB as the dependent variable. The fit of the overall model was set up and tested in SPSS-Amos28 software. The results showed that all the adaptation indexes were within the statistical range, indicating that the mediation model was well fitted (χ^2^/df = 1.495,CFI = 0.957, TLI = 0.940, IFI = 0.959, RMSEA = 0.079).

**Fig. 2:**
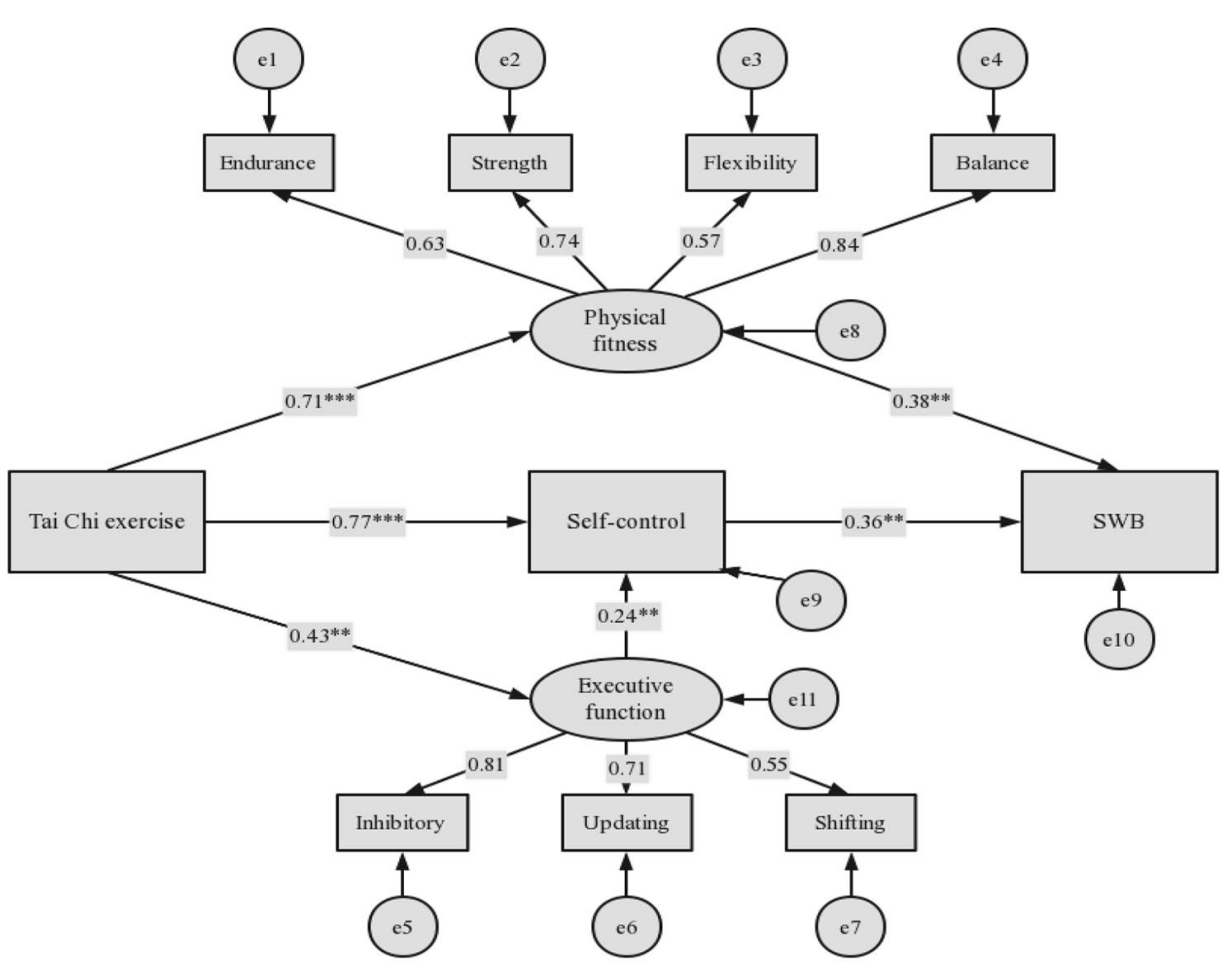
Mediating effect model of Tai Chi exercise and subjective Well-being

According to the results in Fig. 2 (also seen in Table 4), the path coefficients of all influence relationships in this model are significant (ps < 0.001). The Bias-Corrected Bootstrap method was used to verify the mediating and chain mediating effects of physical fitness, self-control and executive function between Tai Chi exercise and SWB, in which the sampling number was set to 2000. The results showed that the indirect effect for path of Tai Chi exercise → physical fitness →SWB was 0.271 (95% CI=[0.115, 0.485], excluding 0), accounting for 46.0% of the total effect. The indirect effect for path of Tai Chi exercise → self-control →SWB path was 0.280 (95% CI=[0.109, 0.433], excluding 0), accounting for 47.5% of the total effect. The indirect effect for path of Tai Chi exercise → executive function → self-control →SWB path was 0.038 (95% CI=[0.009, 0.104], excluding 0), accounting for 6.5% of the total effect.

**Table 4:** Test for mediating effect on Tai Chi exercise and subjective Well-being

	Path	Standardized effect	Standard error	95% confidence interval	
				LLCI	ULCI
Indirect effect	TC→PF→SWB	0.271	0.090	0.115	0.485
	TC→SC→SWB	0.280	0.085	0.109	0.433
	TC→EF→SC→SWB	0.038	0.023	0.009	0.104
Total effect	TC→SWB	0.589	0.067	0.422	0.709

Note: TC = Tai Chi exercise; LLCI = low limit for confidence interval; ULCI = upper limit for confidence interval

## Discussion

In this study, we utilized Tai Chi exercise as an intervention in healthy aged individuals, taking into account age-related changes in physical health (physical fitness) and cognitive function (executive function, self-control). The development of SWB was compared between the Tai Chi intervention group and the group of aged individuals with normal living habits (no exercise). This study verified that Tai Chi intervention has a promoting effect on SWB, cognitive function, and physical fitness in aged people, and also verified that cognitive function and physical fitness play a mediating role in it.

### Influence of Tai Chi exercise on SWB of the aged

This study verified hypothesis 1: Tai Chi exercise has a positive effect on SWB of the aged.

This result is consistent with previous research findings [55–58]. Biological research suggests that this influence may be due to physical exercise increasing gray matter volume in the prefrontal cortex and hippocampal gyrus [59], which reducing gray matter damage [60], and improving cognitive function. In this study, we explored the effects of physical exercise on physical fitness and cognitive function in older adults from an aging perspective, in order to improve their ability to perform activities of daily living, social skills, and functional abilities, ultimately increasing their self-esteem and life satisfaction, and relieving depression and anxiety. Additionally, our research found that participation in physical exercise can improve physical fitness, promote bodily health, and enhance subjective well-being [61]. Biological research has also shown that physical exercise can stimulate the organs of movement, promote bodily health, and enhance the experience of happiness [17]. Therefore, improving the SWB of aged people through Tai Chi exercise is mainly due to changes in their physical fitness and cognitive function.

### The mediating effects of physical fitness and cognition

#### The mediating effect of physical fitness

This study verified hypothesis 2: Physical fitness plays a mediating role in the process of physical exercise affecting SWB.

Firstly, Tai Chi exercise has a notable impact on the physical fitness of the aged, particularly in terms of improving their strength and balance, which is consistent with the results reported by Winter et al. [62] and Tse et al. [63]. Taheri et al. [64] found that aged people showed significant improvement in functional testing and posture control (static balance index and dynamic balance index) after weight bearing exercise intervention.Similarly, Schaller et al. [65] and Wehner et al. [66] also found that the time of standing on one foot with eyes closed significantly increased through Tai Chi exercise in the aged, resulting in a significant improvement in balance.

Weinecke et al. [67] suggested that the unique sequence of movements in Tai Chi exercise may be a related reason for the improvement of muscle strength, balance in aged people. Indeed, the characteristic of Tai Chi is that each action requires the coordination of skeletal and respiratory muscles, requiring multiple muscle groups to participate in completing the action and maintaining body balance, thereby improving the synergistic ability of active, synergistic, antagonistic, and fixed muscles, causing changes in muscle fibers in the aged, thereby improving their strength and balance ability.

Muscle strength and balance are crucial prerequisites and guarantees for the work and life of the aged and are the foundation for production, living, and participation in social activities. Some studies have shown that muscle strength and quality are strongly correlated with longevity and all-cause mortality [68]. Enhancing the strength and balance of the aged can improve their functional ability, increase their life satisfaction and social participation, reduce the risk of falls, and ultimately enhance their subjective well-being.

#### The mediating effect of cognition

This study verified hypothesis 3: Self-control plays a mediating role between Tai Chi exercise and SWB.

Current results has shown that Tai Chi exercise can enhance self-control in the aged, which is consistent with previous research findings [24, 69, 70]. Motor skills are the external manifestation of brain activity, which not only requires the brain to accurately control muscle system, but also involves a large number of cognitive activities, such as perceptual processing, pattern recognition, attention, and decision-making [71]. Tai Chi is unique in that each movement integrates elements of opposition and unity, such as “opening and closing,“ “circle and square,“ “curling and expanding,“ “virtual and real,“ “lightness and heaviness,“ “softness and hardness,“ and “slow and fast.“ Tai Chi emphasizes the natural coordination of breathing and movements, striving to achieve a high degree of unity in mind, strength, energy, and form. It requires individuals to master rhythm, amplitude, strength, and coordination of multiple body parts during the learning process. This frequent stimulation can improve self-control abilities in the aged. Therefore, Tai Chi exercise can not only enhance muscle strength and balance but also promote the development of self-control in the aged.

Additionally, according to the theory of three stages of motor skill learning [72], the initial muscle work of skill formation is characterized by stiff and uncoordinated actions and redundant actions, and then develops to the stage where most wrong actions can be corrected, and individuals can complete actions smoothly and coherently, and finally develop to the stage where individuals can complete all actions accurately, smoothly and elegantly. The above development process is completed under Neuromodulation. The characteristics of its neural activities are that the excitement and inhibitory of the cerebral cortex in time and space are gradually concentrated and then more concentrated and precise, which ultimately leads to the improvement of individual self-control. The internal physiological mechanism is that the brainstem integrates the descending motor instructions of the high-level center with the ascending information of the spinal cord, and then regulates the activity of motor neurons through the brainstem descending pathway to achieve control of movement.

This study verified hypothesis 4: executive function and self-control play a chain mediating role between Tai Chi exercise and SWB.

Furthermore, according to the characteristics of Tai Chi, practicing Tai Chi requires not only memory processing, but also other cognitive participation, such as perception speed, visual spatial ability, attention, multitasking, and planning. Through the activation of limb movement, the activity of the prefrontal cortex of the brain can be stimulated [73, 74], which in turn stimulates the excitation of brain cells, changes the brain’s perception function, and improves the memory of the aged, achieving the effect of brain health [38]. In addition, research has shown that the improvement of executive function is closely related to the structure of the prefrontal lobe. It is possible that during Tai Chi exercise, stimulating the prefrontal cortex can improve the executive function of the aged. In addition, from the perspective of skill development laws, the formation of action skills is also a process of differentiation inhibitory establishment, and also requires good memory and conversion. This shows that Tai Chi exercise can improve the executive function of aged people.

Baumeister et al. [75] found that 80–90% of self-control is related to inhibitory functions, which include resisting desires and impulses, controlling thoughts and emotions, etc. Studies in cognitive neuroscience have also shown that self-control is closely linked to the executive function of the prefrontal lobe [76]. Based on previous researches, Hofmann et al. [77] summarized the relationship between the three subcomponents of executive function and self-control (as shown in Table 5). It can be seen that executive function is an important supportive mechanism for individual self-control in the process of achieving goals. Therefore, after improving the executive function of the aged, Tai Chi exercise affects their self-control development and ultimately affects their SWB.

**Table 5:** Relationship between executive function and self-control

Executive function	Self-control
Updating	Setting self-regulation goals proactively.
	Top-down attention control directs and focuses on goal-relevant information while ignoring irrelevant stimuli.
	Prevent the target standards for self-regulation from being disrupted.
	Translation: Regulating unnecessary emotions, desires, and cravings.
Inhibitory	Passively inhibiting potential impulses, habits, and behaviors.
Shifting	Flexibly shifting between different ways of achieving goals (strategy shifting).
	Translation: shifting between multiple goals (goal shifting).

Note: Adapted from Hofmann et al. (2012) [77]

## Conclusions

1. Tai Chi exercise can promote SWB of the aged;
2. Tai Chi exercise can influence SWB of the aged through the separate mediating effects of physical fitness and self-control;
3. Tai chi exercise can indirectly affect SWB in the aged through the chain mediating effect of executive function and self-control.

### Research significance and limitations

This study explores the mechanism of Tai Chi exercise in enhancing SWB in healthy aged people, and confirms the mediating role of physical fitness and cognitive function between Tai Chi exercise and SWB. The research findings can provide reference for families, communities, aged care institutions, and rehabilitation centers to develop intervention strategies and plans to help the aged to improve their mental health, alleviate negative emotions, enhance self-confidence and independence, improve their sense of well-being, and maintain a positive, healthy, and meaningful life in old age.

However, due to limitations in the research conditions, there are some shortcomings in this study. Firstly, in terms of research design, this study only implemented a 12-week exercise intervention and analyzed data from two time points before and after the intervention. Therefore, the research results can only reveal the effects during the exercise period. Further research could extend the exercise intervention cycle, explore whether the SWB of aged will decrease after stopping exercise intervention, when the turning point of the decline is, and what changes in physical fitness and cognitive function will occur after the intervention is stopped.Second, the sample size of this study was relatively small, the control group did not receive an alternative intervention, there was no blinded assessment, and no longitudinal design was used. Further studies could consider expanding the sample size while setting up a control group to receive an alternative intervention (a different type of physical activity).Finally, there are many variables that affect the SWB of aged, such as attention, self-efficacy, and social relationships, which may also play mediating role between the exercise intervention and SWB. Future research should take these factors into consideration.

## Data Availability

The data that support the fndings of this study are available from the corresponding author H. W. (Heng Wang, email: nmgwangh@163.com), upon reasonable request.

## References

[1] Diener E, Lucas RE, Oishi S (2018). Advances and open questions in the science of subjective well-being. Collabra: Psychol.

[2] Strategy WHOG. Action Plan on Ageing and Health. World Health Organization: Geneva, Switzerland,; 2017.

[3] Diener E, Pressman SD, Hunter J (2017). If, why, and when subjective well-being influences health, and future needed research. Appl Psychology: Health Well‐Being.

[4] Diener E, Lucas RE, Oishi S (2002). Subjective well-being: the science of happiness and life satisfaction. Handb Posit Psychol.

[5] Sin NL (2016). The protective role of positive well-being in cardiovascular disease: review of current evidence, mechanisms, and clinical implications. Curr Cardiol Rep.

[6] Yu LIAO, Zi-chao CHEN (2022). Meta-analysis of the Effect of Physical Exercise on the intervention of Subjective Well-being of the Elderly. SICHUAN SPORTS SCIENCE.

[7] Martela F, Sheldon KM (2019). Clarifying the concept of well-being: psychological need satisfaction as the common core connecting eudaimonic and subjective well-being. Rev Gen Psychol.

[8] Diener E, Seligman MEP (2018). Beyond money: progress on an economy of well-being. Perspect Psychol Sci.

[9] Steptoe A, Deaton A, Stone AA (2015). Subjective wellbeing, health, and ageing. The Lancet.

[10] Taylor BA, Pescatello LS (2016). For the love of it: affective experiences that may increase physical activity participation among older adults. Soc Sci Med.

[11] WANG-FU baihui. Social cause or healthy option? An empirical study on the Health Inequality of Elderly in China. Volume 53. CHINA SPORT SCIENCE AND TECHNOLOGY; 2017. pp. 13–20. 6.

[12] Akbari A, Mirakhori F, Ashouri M et al. The effect of micronutrient intake on cognitive function and physical activity of the elderly. Int J Sport Stud Health, 2021, 4(1).

[13] Vancini RL, dos Santos Andrade M, de Lira CAB (2022). Is it possible to Age Healthy by Performing Ultra-endurance exercises?. Int J Sport Stud Health.

[14] Naghavi N, Taheri M, Irandoust K. Psychophysiological responses to cognitive and physical training in obese elderly. Int J Sport Stud Health, 2018, 1(3).

[15] Taheri M, Irandoust K (2017). The effect of balance exercises and computerized cognitive training on psychomotor performance in elderly. J Phys Therapy Sci.

[16] Taheri M, Irandoust K, Modabberi S (2019). An acute bout of dynamic sitting exercises improves stroop performance and quality of sleep in older adults with cognitive impairment. Int Archives Health Sci.

[17] Yucheng QIAO, Yanzhi FAN (2020). Interrogation and response: eight basic problems in the study of the relationship between physical activity and happiness. J Shanghai Sport Univ.

[18] Hao ZHOU, Qianyu ZHOU (2022). Effect of Physical Exercise on Subjective Well-being of College students: Chain Mediation Effect of Cognitive Reappraisal and Resilience. J Shandong Sport Univ.

[19] Hillman CH, Erickson KI, Kramer AF (2008). Be smart, exercise your heart: exercise effects on brain and cognition. Nat Rev Neurosci.

[20] Chang YK, Nien YH, Tsai CL (2010). Physical activity and cognition in older adults: the potential of Tai Chi Chuan. J Aging Phys Activity.

[21] Zou L, Loprinzi PD, Yu JJ (2019). Superior effects of modified chen-style tai chi versus 24-style tai chi on cognitive function, fitness, and balance performance in adults over 55. Brain Sci.

[22] Zhou S, Zhang Y, Kong Z (2019). The effects of tai chi on markers of atherosclerosis, lower-limb physical function, and cognitive ability in adults aged over 60: a randomized controlled trial. Int J Environ Res Public Health.

[23] Richards J, Jiang X, Kelly P (2015). Don’t worry, be happy: cross-sectional associations between physical activity and happiness in 15 european countries. BMC Public Health.

[24] Buecker S, Simacek T, Ingwersen B (2021). Physical activity and subjective well-being in healthy individuals: a meta-analytic review. Health Psychol Rev.

[25] Chaddock L, Pontifex MB, Hillman CH (2011). A review of the relation of aerobic fitness and physical activity to brain structure and function in children. J Int Neuropsychol Soc.

[26] Huang T, Larsen KT, Ried-Larsen M (2014). The effects of physical activity and exercise on brain‐derived neurotrophic factor in healthy humans: a review. Scand J Med Sci Sports.

[27] Dishman RK, O’Connor PJ (2009). Lessons in exercise neurobiology: the case of endorphins. Ment Health Phys Act.

[28] Li F, Harmer P, McAuley E (2001). Tai Chi, self-efficacy, and physical function in the elderly. Prev Sci.

[29] Fuzhong L, Peter H, Edward MA (2001). An evaluation of the effects of Tai Chi exercise on physical function among older persons: a randomized controlled trial. Ann Behav Med.

[30] Chodzko-Zajko WJ, Proctor DN, Singh MAF (2009). Exercise and physical activity for older adults. Med Sci Sports Exerc.

[31] Manini TM (2010). Energy expenditure and aging. Ageing Res Rev.

[32] Vainionpää A, Korpelainen R, Leppäluoto J (2005). Effects of high-impact exercise on bone mineral density: a randomized controlled trial in premenopausal women. Osteoporos Int.

[33] Lemura LM, Von Duvillard SP, Mookerjee S (2000). The effects of physical training of functional capacity in adults: Ages 46 to 90: a meta analysis. J Sports Med Phys Fitness.

[34] Kelley GA, Kelley KS (2001). Aerobic exercise and resting blood pressure in older adults: a meta-analytic review of randomized controlled trials. Circulation.

[35] Candow DG, Chilibeck PD, Abeysekara S (2011). Short-term heavy resistance training eliminates age-related deficits in muscle mass and strength in healthy older males. J Strength Conditioning Res.

[36] Ramirez-Campillo R, Diaz D, Martinez-Salazar C et al. Effects of different doses of high-speed resistance training on physical performance and quality of life in older women: a randomized controlled trial. Clin Interv Aging, 2016: 1797–804. 10.2147/CIA.S121313 PMC5167493 28008239

[37] Milyavskaya M, Berkman ET, De Ridder DTD (2019). The many faces of self-control: Tacit assumptions and recommendations to deal with them. Motivation Sci.

[38] Zhidong CAI, Shujie LOU, Aiguo CHEN (2021). Expert Consensus on the dose-effect relationship of physical Exercise Delaying Cognitive decline in the Elderly. J Shanghai Sport Univ.

[39] Tu Y, Yang Z (2016). Self-control as mediator and moderator of the relationship between social support and subjective well-being among the chinese elderly. Soc Indic Res.

[40] Duckworth AL, Kern ML (2011). A meta-analysis of the convergent validity of self-control measures. J Res Pers.

[41] Vaughan L, Giovanello K (2010). Executive function in daily life: age-related influences of executive processes on instrumental activities of daily living. Psychol Aging.

[42] Ye M, Wang L, Xiong J (2020). The effect of mind–body exercise on memory in older adults: a systematic review and meta-analysis. Aging Clin Exp Res.

[43] Xiong J, Ye M, Wang L (2021). Effects of physical exercise on executive function in cognitively healthy older adults: a systematic review and meta-analysis of randomized controlled trials: physical exercise for executive function. Int J Nurs Stud.

[44] Persson J, Nyberg L, Lind J (2006). Structure–function correlates of cognitive decline in aging. Cereb Cortex.

[45] Tucker-Drob EM (2011). Global and domain-specific changes in cognition throughout adulthood. Dev Psychol.

[46] Convit A, Douyon R, Yates KF et al. Frontotemporal abnormalities and violent behavior. Mahwah, NJ:Lawrence Erlbaum Associates:Aggression and Violence: Genetics, Neurobiological, and Biosocial Perspectives, 1996: 169–194.

[47] Cauffman E, Steinberg L, Piquero AR (2005). Psychological, neuropsychological and physiological correlates of serious antisocial behavior in adolescence: the role of self-control. Criminology.

[48] Beaver KM, Wright JP, Delisi M. Self-control as an executive function: reformulating Gottfredson and Hirschi’s parental socialization thesis. Criminal Justice & Behavior, 2007, 34 (10): 1345–1361.

[49] Chen T (2009). Development of the subjective well-being scale for the elderly in China. Lan zhou xue kan.

[50] Shen, Zili (2010). Cai Taisheng. Reviston of the General Domain sense and modes of Control Scale. China J Health Psychol.

[51] Li Y (2021). The Effect of High Intensity Intermittent Exercise on executive function of College Students.

[52] Li Zhangping (2019). The impact of aging and exercise on executive function in elderly women.

[53] Peng F (2018). State anxiety and shifting function: examination of the applicability of attention control theory in athletes. J Psychol Sci.

[54] Chong-min JIANG, Dao-zhong YU, Cheng-ye JI (2004). Study of National Physical Fitness evaluation Standard. SPORT Sci.

[55] Wiese CW, Kuykendall L, Tay L (2018). Get active? A meta-analysis of leisure-time physical activity and subjective well-being. J Posit Psychol.

[56] Zhang Z, Chen W (2019). A systematic review of the relationship between physical activity and happiness. J Happiness Stud.

[57] Li Li-jie (2017). Research progress on psychological effects of Taiji exercise. Mod Prev Med.

[58] Pan Y (2016). The impact of Tai Chi Exercise on the subjective wellbeing of Middle and Elderly People from the perspective of healthy aging. J Hubei Normal Univ (Philosophy Social Science).

[59] Erickson KI, Miller DL, Weinstein AM (2012). Physical activity and brain plasticity in late adulthood: a conceptual and comprehensive review. Ageing Res.

[60] Chaddock-Heyman L, Erickson KI, Holtrop JL (2014). Aerobic fitness is associated with greater white matter integrity in children. Front Hum Neurosci.

[61] Huang H, Humphreys BR (2012). Sports participation and happiness: evidence from US microdata. J Econ Psychol.

[62] Winter DA, Patla AE, Prince F (1998). Stiffness control of balance in quiet standing. J Neurophysiol.

[63] Tse S-K, Bailey DM (1992). T’ai chi and postural control in the well elderly. Am J Occup Therapy.

[64] Taheri M, Irandoust K, Moddaberi S (2019). The effects of weight-bearing exercise on postural control and fatigue index of elderly males. Int Archives Health Sci.

[65] Schaller KJ (1996). Tai Chi Chih: an exercise option for older adults. J Gerontol Nurs.

[66] Wehner C, Blank C, Arvandi M (2021). Effect of Tai Chi on muscle strength, physical endurance, postural balance and flexibility: a systematic review and meta-analysis. BMJ open Sport & Exercise Medicine.

[67] Weineck J. Optimales Training: leistungsphysiologische Trainingslehre unter besonderer Berücksichtigung des Kinder- und Jugendtrainings. Optimales Training: leistungsphysiologische Trainingslehre unter besonderer Berücksichtigung des Kinder- und Jugendtrainings, 2009.

[68] Mitchell WK, Williams J, Atherton P et al. Sarcopenia, dynapenia, and the impact of advancing age on human skeletal muscle size and strength; a quantitative review. Front Physiol 2012, 3(1): 260–93. 10.3389/fphys.2012.00260 PMC3429036 22934016

[69] Bae W, Ik Suh Y, Ryu J (2017). Physical activity levels and well-being in older adults. Psychol Rep.

[70] Netz Y, Wu MJ, Becker BJ (2005). Physical activity and psychological well-being in advanced age: a meta-analysis of intervention studies. Psychol Aging.

[71] Wang Shuming (2018). Motor Skill Learning and Control.

[72] Wang, Ruiyuan (2012). Su Quansheng. Exercise Physiology.

[73] Kramer AF, Hahn S, McAuley E (2000). Influence of aerobic fitness on the neurocognitive function of older adults. J Aging Phys Act.

[74] Jiang Changhao Y, Shoufu WS (2012). Physical activity promotes executive function of the brain: behavioral, EEG, and MRI Studies. Chin J Sports Med.

[75] Baumeister RF (2014). Self-regulation, ego depletion, and inhibitory. Neuropsychologia.

[76] LI Jianbin (2013). The mechanism of why self-control resources and cognitive resources influence each other: an Integrated Model. Adv Psychol Sci.

[77] Hofmann W, Luhmann M, Fisher RR (2014). Yes, but are they happy? Effects of trait self-control on affective well‐being and life satisfaction. J Pers.

